# A Systematic Review of Discrete Choice Experiment Studies in Rheumatoid Arthritis Biological Medicines

**DOI:** 10.31138/mjr.32.2.104

**Published:** 2021-06-30

**Authors:** Saman Zartab, Shekoufeh Nikfar, Naeim Karimpour-Fard, Ahmadreza Jamshidi, Vida Varahrami, Ali Homayouni, Abbas Kebriaeezadeh

**Affiliations:** 1 Pharmaceutical Management & Economic Research Center and Department of Pharmacoeconomics and Pharmaceutical Administration, Faculty of Pharmacy, Tehran University of Medical Sciences, Tehran, Iran; 2 Rheumatology Research Center, Tehran University of Medical Sciences, Tehran, Iran; 3 Department of Economics, School of Economics and Political Sciences, Shahid Beheshti University, Tehran, Iran

**Keywords:** Rheumatoid arthritis, discrete choice experiment, patient preference, biological products, conjoint method

## Abstract

**Objective:**

Rheumatoid arthritis is a chronic disease with various clinical characteristics. The introduction of biological drugs has enhanced the efficacy and increased diversity of treatment options. Considering the patients’ preferences in decision-making about treatment can improve their adherence. A discrete choice experiment is a type of conjoint method that can elicit preferences in more realistic scenarios. This article reviewed discrete choice experiment (DCE) studies to extract which attributes and levels were included in surveys. In addition, we focused on the process of designing surveys and the method that they used. Method: PubMed, EMBASE, Web of Science, Scopus, Ovid (Medline) and ProQuest were systematically searched in order to find studies that evaluated rheumatoid arthritis patients’ preferences about biological medicines. Studies published in peer-reviewed journals between 1/1/1990 and 12/31/2019 were included. The included studies were analyzed using a narrative synthesis method and descriptive statistics.

**Results:**

A total of 7124 studies were initially found. After deleting irrelevant and duplicate studies, 15 studies were included. The most common attributes that were used in surveys were efficacy, adverse effect, route of administration, frequency of administration, and cost. Most studies used a literature review for developing attributes and levels. The median number of included attributes and levels were seven and three, respectively. Eight studies explained their experimental design while seven studies did not. Conditional logit and mixed logit were the most common methods for modeling reciprocally.

**Conclusion:**

Several aspects of DCE studies investigating biological drugs in RA were assessed. Explaining the sample size, experimental design, and qualitative work for developing attributes can improve this type of study.

## INTRODUCTION

Rheumatoid Arthritis (RA) is a long-term autoimmune disease characterised by symmetric peripheral joint inflammation. Persistent inflammation can damage cartilage and cause bone erosion.^1^ In addition, RA may present with other clinical features, such as morning stiffness, pain, fever, fatigue, and weight loss. The prevalence of this debilitating disease is 0.5-1.0% in the general population, and is 2–3 times more prevalent in females.^2^

Glucocorticoids, nonsteroidal anti-inflammatory drugs (NSAIDs), and disease-modifying anti-rheumatic drugs (DMARDs) are traditional therapeutic items. DMARDs alter the inflammatory process and reduce associated complications like joint damage. The development of biological agents has had a significant impact on treatment success. Although biological agents are highly effective, they are associated with an increased risk of severe adverse effects, such as life-threatening infections and malignancies.^3^ Because of increased efficacy, adverse effects, and other characteristics (eg, route of administration, frequency, cost, etc.), there are more clinical options for appropriate treatment.

Considering the patients’ preferences in choosing the treatment option can increase their satisfaction with treatment and improve adherence to it. In the long term, patient preference studies can lead to the development of more user-friendly drugs. Previous studies showed differences between the priorities of patients and health care professionals.^4–6^ Decision-making among treatment options in RA patients depends not only on health outcomes, but also on other features of care, such as how and where patients receive them, or the cost of treatment.

There are many different methods to elicit patient preferences directly or indirectly. A discrete choice experiment is an indirect method that is rooted in the random utility theory.^7^ In contrast to asking patients directly to state their preferences, discrete choice experiment studies (DCEs) extract preferences based on choices. The participants are requested to choose their best option among various presented options in a choice task. Each alternative is a combination of multiple attributes with different levels in an orthogonal design. Because of repeated decisions about realistic scenarios in orthogonal and balanced design, this process is less complex (only a subset of levels is given to a participant for each decision). Due to little chance of measurement error, DCE is less prone to violating the statistical assumptions than direct methods.

Although there are some general reviews on the DCE method,^8–10^ only one study focused on patients’ preferences in RA.^11^ They included any method of eliciting preferences, including conjoint analysis, standard gamble, time trade-off, visual analogue scale, and rating or ranking of treatment outcomes. Due to various concepts and designs of the conjoint methods, their results may be different. Thus, we specifically focused on the DCE method and ignored other conjoint analysis methods such as adaptive conjoint analysis, or rank-based full profile conjoint. We considered one more year compared to the previous study and added three articles and three conference abstracts to the DCE part.

This systematic review was conducted on DCE studies investigating biological agents to extract which attributes and levels were used for RA patients. Another objective of this paper was to identify how the attributes and levels were developed for DCE studies. This article also focused on the experimental design of studies, type of modeling, and the method of developing and presenting questionnaire.

## METHODS

### Study design and data sources

**Figure 1.**
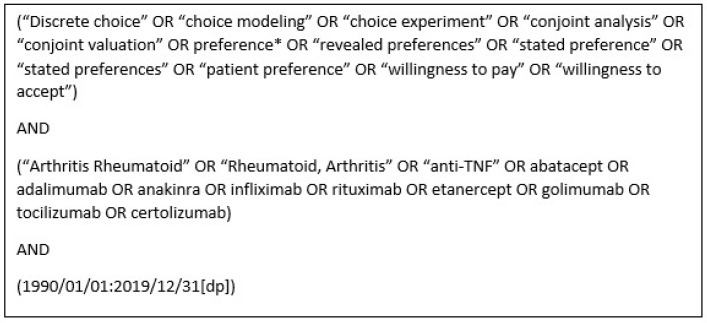
Search strategy used for PubMed.

A comprehensive search strategy was developed for searching PubMed, EMBASE, Web of Science, Scopus, Ovid (Medline) and ProQuest databases. The search components were Rheumatoid Arthritis and its synonyms; discrete choice experiment, its synonyms, and more general concepts (eg, conjoint analysis, patient preferences); biological DMARDs, and the specific names of biological drugs related to RA. The search time interval was from 1/1/1990 to 12/31/2019. Two members of the team (SZ and NK) found synonyms for search components via searching the MeSH and Emtree and interviewing with two experts. Before running search strategies, they were checked using the PRESS checklist. **Figure 1** illustrates the search strategy for PubMed.

### Study selection

**Figure 2.**
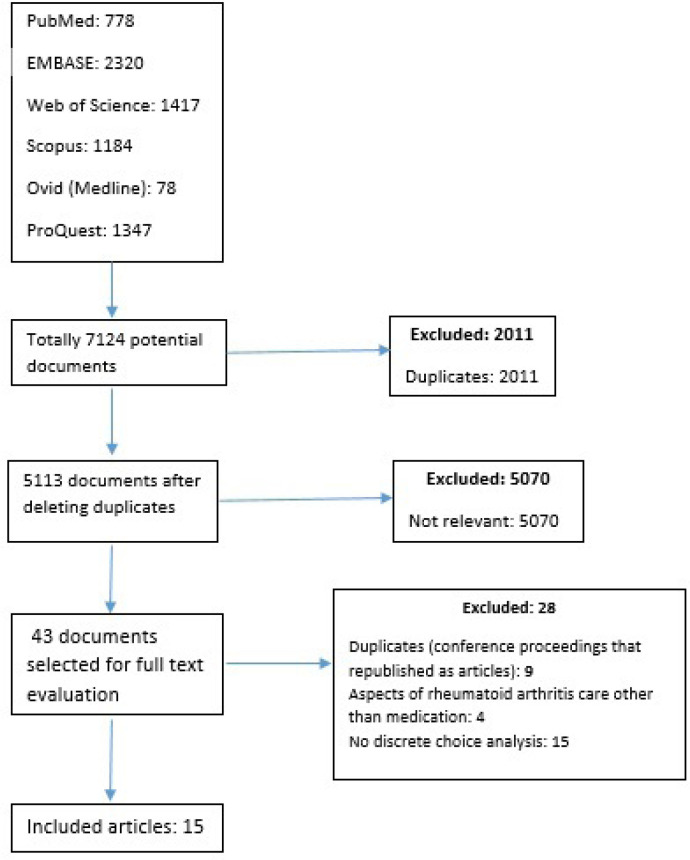
PRISMA flow diagram illustrating study selection.

The search results were saved in the Endnote library. The duplicate studies were identified and removed from the Endnote library. Two authors (SZ and NK) reviewed the abstracts and selected the studies for full-text review. At the end of the search process, the reference lists of the included studies were searched manually. The search process is demonstrated in **Figure 2**.

### Inclusion and exclusion criteria

Only studies that were published in peer-reviewed journals were included. The studies that elicited the preferences of RA patients (18≥ years) for biological DMARDs were included. The patients with any race or ethnicity, gender and occupation were included. Studies that only looked at rheumatologists or other healthcare professionals’ preferences were excluded. Studies that compared the preferences of healthcare professionals with patients were excluded, unless the patients’ data could be extracted separately. Studies using the DCE method were included and other conjoint analyses like ACA, rating, or ranking methods were excluded. The included articles were original articles or conference abstracts. Other types of studies like reviews, commentaries, editorials, opinions, and letters were excluded. Studies with any sampling method (random or non-random) and any sample size were included. Disputes were resolved by consensus or the judgment of the corresponding author.

### Data extraction and analysis

**Table 1** illustrates the included documents for final analysis. Two reviewers extracted the data according to a form that was developed for focusing on study design features, attributes, and levels. The extracted data included the study method, study place, sample size, funding sources, data modeling method, experimental design, number of choice tasks per questionnaire, method of running the survey, data analysis software, method of choosing and developing attributes and levels, number of attributes and levels, and method of presenting information in choice tasks. Quality assessment of the studies was done by two reviewers (SZ and NK) using a checklist for the quality of cross-sectional studies (AXIS).^13^ A narrative synthesis method was used to analyse the included studies.^14^ Statistical analysis was conducted using Excel 2013.

**Table 1.** Included studies and some of their characteristics.

**Study**	**Country**	**Sample size**	**Analysis**	**No. of attributes**	**Mean levels per attribute**	**Article type**	**Year**
Alten et al.^1^	Germany	1588	Best-worst-scaling	5	2.8	Original article	2016
Augustovski et al.^15^	Argentina	240	Multinomial probit regression model (MNP)	7	3	Original article	2013
Diaz et al.^16^	Spain	137	Conditional logit model	7	NA 2-4	Conference Abstract	2018
Harrison et al.^17^	Canada	78	Conditional logit model and a mixed logit model	5	NA	Conference Abstract	2018
Hazlewood et al.^18^	Canada	152	Multinomial logit model	8	3	Original article	2016
Husni et al.^3^	USA	510	Multivariable Logistic regression model	9	3.3	Original article	2017
Louderet al.^19^	USA	380	Hierarchical Bayes model	7	3.3	Original article	2016
Nafees et al.^20^	UK,USA	287	Conditional logit model	6	2.5	Conference Abstract	2012
Poulos et al.^21^	USA	901	Mixed-logit methods	6	3.3	Original article	2014
van Heuckelum et al.^22^	Netherlands	325	Latent class analysis and multinomial logistic regression	7	3	article	2019
Scalone et al.^23^	Italy	513	Random-effects conditional logistic regression model	6	2.8	Original article	2017
Ho et al.^24^	Australia	206 (85)*	Restricted latent class model (LCM)	8	2.8	Original article	2019
Fraenkel et al.^25^	USA	1273	Latent class Analysis	7	3.1	Original article	2017
Özdemir et al.^26^	USA	466	Mixed logit	6	3.8	Original article	2009
Skjoldborg et al.^27^	Denmark	178	Random effect logit model	6	6.7	Original article	2009

* Number of RA patients in the study.

## RESULTS

### Study participants

**Table 1** demonstrates the country where each study was carried out. Eight studies were conducted in North America (6 studies in the USA and 2 in Canada). Six studies, one of which was common with the USA were done in Europe (Germany, Spain, the UK, the Netherlands, Italy, and Denmark). Two studies were conducted in Argentina and Australia.

### Diagnosis

Thirteen studies only included RA patients. Two articles included several types of rheumatoid diseases and RA patients were part of their participants. Twelve studies only investigated patients’ preferences and three studies evaluated the patients’ and health care professionals’ preferences.

### Development of attributes and levels

Most of the studies used a literature review for developing attributes and levels (73.3%). Some studies used qualitative methods to learn the views of RA patients (46.6%) and a number of studies conducted focus groups (40.0%). One study used a nominal group technique for eliciting the ideas of patients and rheumatologists. The majority of the studies used interview as a refiner tool rather than a basis for developing attributes. Two studies (13.3%) did not report the method of choosing and preparing attributes and levels.

### Survey Design

**Figure 3.**
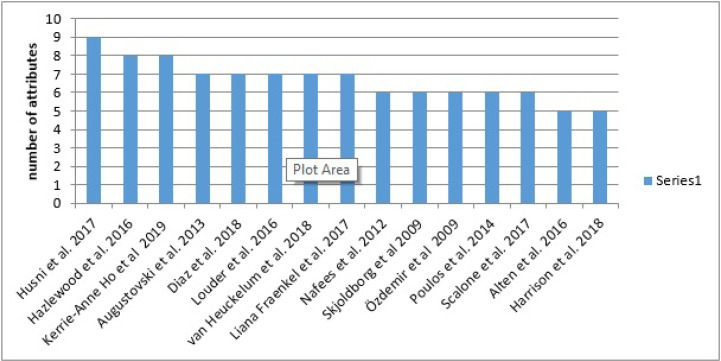
Number of attributes in each study.

**Figure 3** presents the number of attributes that each survey included. The median number of attributes used in surveys was seven, which is in accordance with other “attribute-based stated preference” studies in health economics.^9,10,28^ The maximum number of attributes was 9 and the minimum number of attributes was 5. The median number of levels for characters was 3. The maximum number of levels was 18 and the minimum was 2. One study (a conference abstract) did not report the number of levels for each attribute.

Eight studies reported the type of design they used (fractional factorial, D-optimal). Seven studies did not explain their experimental design (all conference abstracts and two articles). Six studies used the criterion D-efficiency. Four studies applied the Sawtooth software for constructing the experimental design. Four studies used other statistical programs (SAS, spss-11-0, Ngene), and 1 study used the Fedorov algorithm to build its design. Only 2 of 13 surveys reported an opt-out option. Six studies included a dominant option for excluding unconscious participants.

**Figure 4.**
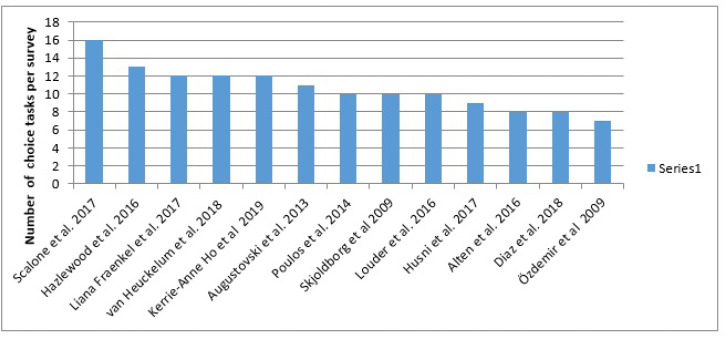
Number of scenarios per survey.

A matter of debate in constructing a DCE questionnaire is how many choice tasks should be presented to the participants without burdening them.^29^ **Figure 4** demonstrates the variety of survey length reported as the choice task per survey in each study. Three studies did not report the number of choice tasks per subject. The median number of choice tasks was 10, with a standard deviation (SD) of 2.4.

**Figure 5.**
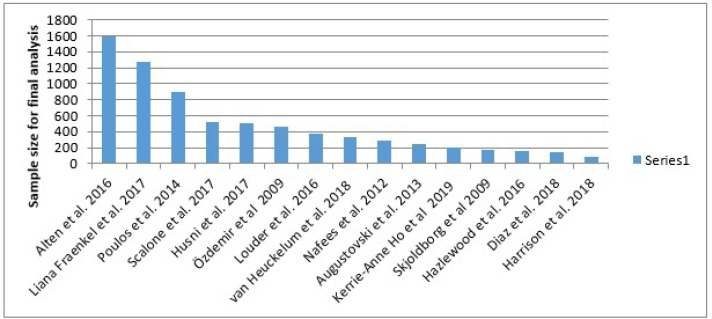
Sample size included for final analysis.

**Figure 5** demonstrates the sample sizes applied in the final analysis. There was a huge variation in the sample size between studies. The median number of participants was 325. Only six studies explained how they selected their sample sizes. Three studies chose sample size base on the prevalence of RA in the population. Three studies explained that they calculated the sample size using the “rule of thumb” and other DCE studies.

Seven studies were conducted online and 3 studies used pens and papers for data collection. Four studies did not report how they gathered the data.

### Attributes

The attributes used in each survey were gathered and categorized in 16 sub-groups and five groups by one of the authors (SZ). All attributes were categorized in at least 1 group and few were placed in 2 groups. Attributes that described efficacy were the most frequent, and the second common attributes were those describing severe side effects. The third and fourth frequent attributes were those describing route of administration and frequency of administration, respectively (**Table 2**).

**Table 2.** Frequency of attributes and number of levels used in included studies.

**Variable**	**Levels**					**Total number of times reviewed in studies**
	Five or more	Four	Three	Two	N/A	
Efficacy (all aspects)	3	6	3	4	3	19
Adverse effects (all aspects)			11	4	2	17
Route of administration	1		5	1	3	10
Frequency of administration	2	4	1	1	1	9
Cost (all aspects)	2		2	1	2	7

### Probability

Risks and benefits are probabilistic phenomena. Most of the studies explicitly reported that they used quantified probability for explaining risks or benefits (11 of 15 [73.3%]).

Three studies reported that they used visual tools like risk grid to demonstrate probabilistic attribute-levels. No study reported the influence of the presentation approach on preferences.

### Analysis Methods

**Table 1** shows the analysis method used in each study. The most frequent model was conditional logit (6 studies) followed by mixed logit (5 studies) and latent class analysis (3 studies). Four studies used the STATA and 4 studies used the Sawtooth software to create models and analyse the data. Two studies reported that they used the R software and 2 studies used the NLOGIT. One study reported that all analyses were conducted in SAS. Five studies did not identify the software they used for data analysis.

## DISCUSSION

Fifteen DCE studies in the field of rheumatoid arthritis were evaluated. All studies investigated the preferences of patients with rheumatoid arthritis for biological medicines. A few studies used qualitative methods for developing attributes and levels, which reflects the need for more attention to developing attributes and documenting this process. RA patients are a heterogeneous population, so involving patients in developing attributes is vital to avoid omitting some important aspects of treatment. The majority of the RA patients are not medical professionals, and some of them suffer from cognitive impairment. Qualitative interviews help to find the most meaningful way to express the attributes. For example, although the DAS-28 is a useful tool for measuring disease activity and a vital criterion for decision-making about the effectiveness of therapy, it is difficult to include such measures in a survey. Thus, a qualitative search is a useful tool for simplifying and developing attributes.

The design of a DCE is vital for judging its results.^29,30^ Many studies did not report the criterion they used for constructing the design of the survey. Some studies did not mention the software they used. Each software has a specific algorithm for design construction.^31^ Thus, it is crucial to mention the software and its version for study reproducibility.

Only 2 studies used an opt-out option. Some patients may prefer no options, although this situation is unrealistic in routine practice. In addition, an opt-out option may result in losing some information, because some respondents choose it to avoid the burden of making difficult decisions.^32^ However, studies that only offer force choices should explain its impact on the results. Future surveys should present an alternative way to offer options like dual-response design in which the participants first decide on a forced-choice and then an opt-out option is presented to them.

There was a wide range of sample size in the reviewed studies, so it was difficult to interpret whether or not a suitable sample size was selected. There are some guides and a “rule of thumb” for calculating the DCE sample size.^33–35^ If the appropriateness of the sample size is unknown, the validity and quality assessment of the study may be associated with some problems. Thus, reporting sample size calculation by an explicit or less formal method is a necessity for future studies.

The attributes related to outcomes and side effects are probabilistic in nature. The preference of people for probabilistic attributes is highly heterogeneous. Some studies presented the benefits and outcomes as certainties causing problems in the external validity of results. Sometimes it is difficult for patients to understand probabilistic attributes, so some studies presented them as deterministic attributes to improve the respondent’s comprehension. On the other hand, there is evidence that different ways of presenting probabilities can affect one’s perceptions.^36,37^ Using graphs or pictographs can improve the participants’ understanding. However, some of the included studies did not use probabilistic attributes, and only 3 of them presented probabilistic attributes visually. No study examined how the mode of presentation influenced the choices.

### Limitations

Our work has some limitations. Due to heterogeneity of results and different methods for modelling, we could not summarise the results quantitatively. In addition, most studies were funded by pharmaceutical companies that may have affected the objective of studies. If there were a variety of funders, it would have increased the diversity of results. Focusing on technical details of discrete choice experiments in RA is a strength of our review, because it can be used as a practical guide for future studies.

## CONCLUSION

Involving patients in the decision-making process is becoming a trend, specifically in chronic diseases such as RA with an evolving drug pipeline. Thus, it is necessary to review the RA patients’ preferences about various treatment options. DCE studies are becoming popular in the field of measuring preferences. We reviewed DCE studies in the field of RA and evaluated several aspects of this type of study. Efficacy, adverse effects, and route of administration were the most frequent attributes included in surveys. Presenting probabilistic attributes in picto-grams and qualitative work for developing attributes can improve the participants’ comprehension in future studies. In addition, describing the method of sample size calculation can aid in assessing the quality of the study.
